# Middle ear cholesteatoma: biological basis, invasiveness heterogeneity, and challenges in severity assessment

**DOI:** 10.1007/s00405-026-10666-2

**Published:** 2026-10-13

**Authors:** Qingqing Yao, Zhengang Zeng, Zhongxing Hu, Jun Wang

**Affiliations:** Department of Otolaryngology-Head and Neck Surgery, Peking University First Hospital, Beijing, 100034 China

**Keywords:** Middle ear cholesteatoma, Bone erosion, Biological aggressiveness, Invasiveness heterogeneity, Severity grading

## Abstract

**Purpose:**

Middle ear cholesteatoma (MEC) is a benign but biologically aggressive middle ear lesion characterized by progressive and destructive bone erosion. However, current evaluation systems remain heterogeneous and focus on limited anatomical structures, leading to insufficient clinical predictive value. This review aims to integrate current evidence on the biological mechanisms of MEC-associated bone erosion and assessment systems, with the goal of informing the development of more standardized and clinically meaningful severity evaluation models.

**Methods:**

A narrative review was performed in PubMed, Web of Science, and Embase databases to summarize current knowledge on the mechanisms driving bone erosion in MEC and to evaluate the existing staging systems for assessing erosion severity. Keywords included “cholesteatoma”, “bone erosion”, “bone resorption”, “molecular mechanisms”, “staging systems” and related terms.

**Results:**

Available evidence indicates that bone erosion in MEC arises from the interaction between hyperproliferative epithelium, chronic inflammation, and osteoclast activation mediated by cytokines and signaling pathways, particularly RANKL-related mechanisms. Existing staging systems are largely descriptive and exhibit significant heterogeneity, which collectively undermines their clinical utility in guiding surgical management or predicting patient outcomes.

**Conclusion:**

Current staging systems, valuable for describing anatomical extension, fail to capture cholesteatoma’s biological heterogeneity and invasive potential, thus incompletely depicting the disease. Future evaluation strategies should integrate molecular mechanisms, comprehensive anatomical involvement, and imaging findings to develop more standardized and clinically meaningful severity assessment systems.

## Introduction

Middle ear cholesteatoma (MEC) is a locally invasive but histologically benign lesion characterized by abnormal proliferation of keratinizing squamous epithelium within the temporal bone, with an incidence of 9–15 per 100,000 adults [1–3]. MEC exhibits a distinctive capacity for progressive and destructive bone erosion, which differentiates it from other chronic inflammatory middle ear diseases. This “bone-invasive” behavior frequently compromises critical anatomical structures, leading to hearing loss, facial nerve dysfunction, vestibular impairment, and potentially life-threatening intracranial complications. Although surgical excision is the primary treatment, long-term recidivism, encompassing both recurrent and residual disease, remains a major clinical challenge, as cumulative recurrence rates continue to rise over time, reaching up to approximately 40% at 15 years postoperatively [4]. Accumulating evidence indicates that MEC-associated bone destruction is not a passive mechanical process but represents an active pathological bone remodeling disorder. At the molecular level, this process is driven by sustained osteoclast activation mediated by receptor activator of nuclear factor-κB ligand (RANKL) signaling [5, 6], excessive degradation of extracellular matrix by matrix metalloproteinases (MMPs) [7], and a chronic proinflammatory microenvironment dominated by cytokines such as interleukin-1β (IL-1β) and tumor necrosis factor-α (TNF-α), which collectively activate NF-κB or EGFR signaling pathways and amplify tissue invasion [8, 9]. These findings collectively redefine MEC bone erosion as a biologically driven process, suggesting that molecular intervention may serve as an adjunct to complement surgical management.

A critical yet underappreciated feature of MEC is the marked heterogeneity in bone erosion severity among patients. Clinically, MEC demonstrates substantial heterogeneity in bone-destructive capacity, ranging from limited ossicular erosion to extensive skull base involvement. Such variability stems from not only intrinsic molecular and inflammatory activity but also differences in immune microenvironment dynamics, biomechanical stress distribution, ventilation status, and microbial exposure [10, 11]. Given the multifactorial nature of bone erosion, its heterogeneity should be understood as the product of complex interactions rather than isolated factors, emphasizing the need for a comprehensive assessment framework to better predict clinical progression.

However, contemporary severity grading systems show insufficient reliability in predicting disease progression, recurrence risk, and long-term outcomes [12, 13]. The heterogeneity of assessment frameworks not only impedes cross-study comparability but also restricts accurate risk stratification and optimization of surgical strategies. In this review, we summarize current evidence regarding the biological mechanisms driving bone erosion in MEC, and further discuss the heterogeneity of invasive behavior and examine existing assessment systems used to evaluate disease severity. By integrating mechanistic insights with current classification frameworks, this review aims to highlight key limitations of current evaluation approaches and provide perspectives for the development of more biologically grounded and clinically applicable severity assessment models.

## Pathological bone remodeling network model of MEC

Existing evidence indicates that MEC manifests as a chronic epithelial proliferative disorder typified by persistent inflammatory signaling, immune activation within the microenvironment, and abnormal osteoclast regulatory control, particularly the dysregulation of the RANKL/RANK/OPG axis, which functions as a core regulatory mechanism [5, 14]. In this framework, bone erosion is not merely a secondary structural complication but reflects a system-level shift toward persistent osteolytic potential accompanied by the continuous generation of MMPs and pro-inflammatory cytokines [15]. Recent progress in the application of single-cell transcriptome profiling has begun to elucidate the intricate cellular microenvironment of MEC [16, 17]. These efforts have identified a specific subset of activin A-producing fibroblasts that collaborate with osteoclastogenic signals to facilitate local bone destruction. Thereby emphasizing a previously unacknowledged function of non-immune stromal cells in inducing osteolysis. Moreover, the established importance of immune microenvironment remodeling in cholesteatoma supports the notion that polarized macrophage states and other immune subsets further modulate osteolytic potential [18]. M1-type macrophages predominate in early lesions, secreting pro-inflammatory cytokines such as IL-1βand TNF-α, which facilitate inflammatory responses and bone destruction. Through the inhibition of TNF-α, the destructive M1 pathway may transition to the homeostatic M2 pathway, potentially guiding the development of biologically targeted therapies for cholesteatoma.

The pathological bone remodeling network model of MEC demonstrates that its biological aggressiveness originates from a self-amplifying loop. This loop integrates epithelial hyperproliferation, immune-mediated inflammatory cascades, and extracellular matrix degradation. As a result, it maintains a chronically activated osteolytic microenvironment and makes bone erosion a continuous and self - sustaining process. Consequently, comprehensively dissecting this pathological network can help understand disease progression and identify novel therapeutic targets, laying the groundwork for pharmacological interventions to disrupt core feedback loops driving disease progression.

## Common patterns of bone erosion and factors influencing erosion severity

The growth characteristics and bone erosion patterns of MEC depend largely on its site of origin and etiology. These features differ significantly across subtypes. It is imperative to acknowledge that there is considerable heterogeneity in the biological behaviors of these subtypes, which impacts their clinical manifestations and progression. A comprehensive comparison of these variations is provided in Table 1, offering deeper insights into the distinct erosion mechanisms and growth patterns.

### Growth pattern-specific erosion characteristics

Based on etiology, MEC is classified into congenital cholesteatoma (CC), primary-acquired cholesteatoma (PAC), secondary-acquired cholesteatoma (SAC) and unclassifiable types (cholesteatomas of uncertain origin), each exhibiting distinct erosion profiles [19]. Studies have shown that Attic-type PAC is characterized by local invasion, extending along the attic to the sinus tympani and mastoid, representing approximately 34.3% of cases, while Tensa-type PAC extends medial to the malleus-incus complex toward the sinus tympani and facial recess, accounting for 33.8% of cases [20]. And pars tensa cholesteatoma exhibits higher facial nerve canal dehiscence rates than pars flaccida disease [21], likely due to its growth along the tympanic segment of the facial nerve.

From the anatomical involvement of bone erosion, ossicular chain involvement represents the most frequent manifestation of cholesteatoma-induced bone erosion. Among the three ossicles, the incus is most susceptible to erosion, followed by the stapes, with the malleus being least affected [22, 23]. This hierarchical vulnerability reflects the anatomical positioning of the ossicles relative to cholesteatoma growth pathways. Facial nerve canal dehiscence occurs in 25.7%–64% of cholesteatoma patients, compared with 9.5%–27.7% in non-cholesteatoma chronic otitis media, and the tympanic segment is the most commonly affected site [24]. Notably, adults demonstrate significantly higher dehiscence rates (25.7%–29.5%) than children, and cases of revision surgery demonstrate a heightened prevalence [25]. These findings indicate that bone erosion in MEC typically begins with ossicular involvement and may extend to the facial nerve canal, labyrinth, or tegmen in a pattern influenced by anatomical origin, ventilation status, and biological aggressiveness. Clinically, individualized surgical strategies should be formulated based on lesion origin and growth characteristics. Precise lesion clearance and anatomical repair can optimize treatment outcomes and improve patient prognosis.

**Table 1:** Comparison of growth patterns and bone erosion characteristics among different types of MEC

Classification	Origin site	Main growth patterns	Bone erosion patterns
Congenital Cholesteatoma	Anterosuperior quadrant	Posterior superior quadrant to the attic to the mastoid	Late mastoid involvement; labyrinth in advanced disease
Primary-Aquired Cholesteatoma(Attic cholesteatoma)	Pars Flaccida	Attic to tympanic sinuses and mastoid	Scutum, followed by the incus and malleus; stapes usually spared
Primary-Acquired Cholesteatoma(Tensa or posterior mesotympanic)	Posterosuperior quadrant of the pars tensa	Mesotympanum to posterior tympanum to inner ear	Incus long process and stapes, followed by the malleus
Secondary-Acquired Cholesteatoma	Secondary to tympanic perforation	Perforation site to tympanic cavity	Extensive ossicular and adjacent bone erosion

### Factors influencing bone erosion severity

Molecular determinants of erosion severity have been increasingly characterized. Parathyroid hormone-related protein (PTHrP) and RANKL expression in cholesteatoma epithelium correlates positively with bone resorption degree [26], suggesting that epithelial cells actively modulate the bone microenvironment through paracrine signaling. The expression of MMP-14 exhibits a notable positive correlation with the severity of bone destruction, regardless of age, gender, or the degree of hearing loss [7]. Keratinization degree has emerged as a critical intrinsic factor. Feng et al. demonstrated that higher keratinization correlates with more severe bone destruction, with keratin layer thickness and Keratin 10 expression showing positive associations with erosion severity. Study confirmed that keratinocytes directly promote monocyte differentiation into osteoclasts, establishing a mechanistic link between epithelial characteristics and bone-resorptive capacity [27]. Studies on the mRNA m⁶A modification profile in MEC have identified a set of differentially modified genes closely related to the Hippo and p53 pathways, which can serve as molecular biomarkers for evaluating the invasiveness and bone destruction severity of cholesteatoma, and be used for preoperative evaluation and prognostic judgment [28]. Clinical and temporal factors also influence erosion patterns. Disease duration exceeding 5 years significantly increases the risk of facial nerve canal dehiscence, highlighting the importance of early intervention [25]. Microbial factors may amplify bone destruction. Metagenomic next-generation sequencing revealed that as pathological severity worsens, microbial diversity increases [11]. In conclusion, understanding molecular, clinical, and microbial factors driving bone erosion in cholesteatoma can help develop targeted therapeutic strategies. Early treatment based on these factors may reduce severe complication risk and improve patient outcomes.

## Evolution and limitations of bone erosion assessment systems in MEC

Accurate assessment of bone erosion is essential for surgical decision-making and prognostic evaluation in MEC. Upon tracing the chronological progression of cholesteatoma staging systems, it becomes apparent that the initial ossicular chain assessment system has evolved into a more quantitative evaluation methodology, with its scope expanding to multiple structures. A comparative summary of staging systems for MEC is presented in Table 2. However, persistent constraints, such as a limited dimensional scope and observer dependency, continue to impede their clinical reproducibility and research applicability.

1. Establishment of Ossicular Chain Assessment Systems
  The Austin-Kartush Classification System (AKCS), originally proposed by Austin in 1971 and subsequently extended by Kartush, remains a foundational framework for assessing ossicular chain damage in chronic otitis media and cholesteatoma [29, 30]. It focuses on the presence/absence and fixation status of the malleus, incus, and stapes. It also noted that malleus presence predicts better postoperative hearing outcomes regardless of stapes condition. Patients with an intact malleus achieve mean air-bone gaps 4–5 dB better than those without. However, the AKCS has significant limitations that restrict its utility for comprehensive bone erosion assessment. First, the binary (present/absent) categorization fails to capture intermediate stages of partial erosion. Second, the system excludes evaluation of non-ossicular structures: facial nerve canal, tegmen tympani, and bony labyrinth, thereby limiting its ability to predict complication risks or reflect overall disease severity.
  Meyerhoff and Truelson published the first comprehensive pathophysiology-based cholesteatoma staging system in 1986 [31]. The evaluation dimensions include pathophysiology, location, eustachian tube function, ossicular defects, and presence of complications. This system is the first attempt to integrate cholesteatoma’s etiology, anatomy, and clinical outcomes into a unified framework, laying the conceptual foundation for the subsequent development of a multidimensional staging system. And the Potsic staging system was specifically designed for congenital cholesteatoma, classifying disease into four stages based on ossicular involvement and mastoid extension in 1993 [32].
2. Quantitative Assessment and Multi-Structural Expansion
  The Maresh scaled rating system, proposed in 2011, was developed to address the fundamental limitation that previous ossicular assessments provided minimal information to describe and quantify cholesteatoma growth patterns [23]. The system employs a 4-level grading scale for each ossicle and a 3-digit code represents the overall ossicular status, significantly improving the granularity of destruction assessment and facilitating standardized documentation for pre/postoperative comparison and multicenter data recording. Validation study demonstrated that the status of each ossicle was significantly associated with air-bone gap in a graded and independent manner, with the incus showing the most significant association [33]. Critically, the system has been adopted in biomarker research, with studies demonstrating that periostin, TNF-α, and IL-6 expression increased progressively with Maresh-graded ossicular destruction severity [34], validating its utility for correlating molecular mechanisms with clinical phenotypes. However, while 64 theoretical codes exist, only a subset are clinically common, and the complex coding scheme increases the learning curve, limiting its widespread adoption. Similar to the AKCS, the Maresh scaled rating system also adheres to an ossicle-centric logic, failing to comprehensively reflect the overall extent of cholesteatoma invasion or the potential risk of complications.
  In 2011, Black and Gutteridge put forward a classification for acquired cholesteatoma based on clinical presentation, categorizing cholesteatomas into three common types: the attic type, the pars tensa type, and the combined type [35]. This classification emphasized differences in pathological features, management strategies, and prognosis among different cholesteatoma types, with combined type showing the worst prognosis. In 2015, the Japan Otological Society (JOS) issued a staging system for MEC, which served as Japan’s first national standardized staging framework [36], laying the foundation for the subsequent EAONO/JOS joint consensus. In light of escalating clinical demand for complication risk assessment, Aslıer et al. developed the Bone Erosion Score (BES) based on the AKCS in 2016 [37]. Based on intraoperative observations, if an ossicle is identifiable despite partial destruction, it gets a score of “1”; if completely destroyed and no longer clearly discernible, it gets a score of “2”. Each item is scored independently and scores are aggregated for a total erosion score. The BES systematically integrates key complication-associated structures for the first time, providing a foundation for warning of intracranial and facial nerve complication risks. Also, a significant positive correlation with the Austin-Kartush classification was confirmed, validating its clinical utility.
3. Multi-Dimensional Comprehensive Staging
  EAONO/JOS Staging System
  The European Academy of Otology and Neurotology (EAONO) and Japan Otological Society (JOS) officially released their joint consensus staging system in 2016, representing the first internationally recognized cholesteatoma staging standard [19]. The system classifies cholesteatoma into four stages, stage I: cholesteatoma confined to a single site; stage II: cholesteatoma involving two or more sites; stage III: extracranial complications (facial paralysis, labyrinthine fistula, postauricular fistula/abscess); Stage IV: itracranial complications. The system received support from the international otological community. International validation involving 9 centers and 1,482 cases demonstrated high inter-rater and intra-rater reliability [38]. Nevertheless, staging disparities were observed in over 10% of the cases, and no database encompassed all the parameters necessary for precise staging.
  STAM/STAMCO Classification
  The STAM/STAMCO classification was proposed in 2017, emphasizing detailed anatomical site mapping. Specifically, S represents the disease site (S1: supratubal recess; S2: sinus tympani); T represents the status of the tympanic membrane; A represents attic involvement; M represents mastoid involvement. The STAMCO classification is an extended STAM version, adding C (Complications) and O (Ossicular chain status) [39]. Comparative investigations indicated that the STAMCO classification exhibited a significant superiority over the EAONO/JOS system in the prediction of recurrent and residual cholesteatoma [40].
  ChOLE Classification
  In 2019, Linder et al. put forward the ChOLE classification as an alternative to the EAONO/JOS system [41]. This classification integrates four dimensions: Ch, which represents the extension range of cholesteatoma; O, denoting the postoperative status of the ossicular chain (O0-O4); L, referring to complications; and E, indicating the function of the eustachian tube and the degree of pneumatization/ventilation. The unique features of this system include assessment of postoperative rather than preoperative ossicular chain status and incorporation of middle ear ventilation status as a prognostic factor.

**Table 2:** Comparative summary of staging systems for MEC

Year	Classification System	Primary Assessment Dimensions	Key Features
1971	Austin Classification	Malleus handle, stapes arch	Only ossicular status
1986	Meyerhoff Staging	Etiology, location, eustachian tube function, ossicular defects, complications	First comprehensive pathophysiology-based staging
1993	Potsic Staging	Ossicular involvement, mastoid extension	Applicable only to congenital cholesteatoma
1994	Austin-Kartush Classification	Ossicular presence/absence/fixation (groups A-F)	Extended Austin system with fixation groups
2011	Maresh Grading	Three-digit ossicular grading	Quantified ossicular erosion degree
2011	Black Classification	Attic, pars tensa, combined types	Clinical presentation-based acquired cholesteatoma classification
2015	JOS Staging	Anatomical extension range	Useful for surgical staging but does not quantify bone destruction
2016	BES (Bone Erosion Score)	Ossicular chain + non-ossicular structures	Quantifies ossicular and adjacent bony erosion
2016/2017	EAONO/JOS Staging	Anatomical extension (Stages I-IV) + complications	International standardized staging; biological aggressiveness not assessed
2017	STAM Classification	S (site), T (tympanic membrane), A (attic), M (mastoid)	Site-based classification
2018	STAMCO Classification	STAM + C (complications) + O (ossicular chain)	Expanded STAM with ossicular and complication assessment
2019	ChOLE Classification	Ch (extension), O (postoperative ossicles), L (complications), E (ventilation)	Comprehensive multidimensional classification; relatively complex

## Comparative prognostic utility of current cholesteatoma staging systems

Comparative evaluations of contemporary cholesteatoma staging systems have yielded heterogeneous conclusions regarding their prognostic value and correlation with clinical outcomes. In a cohort of 231 adult patients, Van der Toom et al. reported that the STAMCO classification demonstrated superior predictive performance for recurrent cholesteatoma compared with the STAM and EAONO/JOS systems [40]. Consistently, Fermi et al. observed that early disease stages defined by STAM stage 1 and EAONO/JOS stage 1 were associated with markedly lower recurrence rates, 0% and 2.6%, respectively. However, within their endoscopic surgery cohort, neither the ChOLE classification nor ossicular chain status or complication grading showed significant prognostic value for predicting residual or recurrent disease [42]. In contrast, Eggink et al. identified no significant association between JOS, EAONO-JOS, or STAMCO staging and disease recidivism, highlighting follow-up duration, surgical technique, and patient age as major confounding factors that hinder development of a universal classification [43]. A recent comparative analysis by Aydin Sudabatmaz et al. further demonstrated that existing systems exhibit differential strengths: the ChOLE classification correlates more closely with intraoperative structural damage, whereas EAONO/JOS staging shows stronger associations with audiological outcomes. Notably, neither system demonstrated robust predictive performance for recurrence [12]. Taken together, these findings suggest that current staging frameworks primarily capture distinct dimensions of disease burden: anatomical destruction, hearing impairment, and surgical findings, rather than providing a unified prognostic model. This limitation underscores the need for integrative staging strategies. The key characteristics and comparative features of these staging systems are summarized in Table 3.

**Table 3:** Comparison of major staging systems

Feature	EAONO/JOS	STAMCO	ChOLE	Potsic
Core dimension	Extension + complications	Anatomical sites + ossicles	Extension + ossicles + ventilation	Ossicular involvement + mastoid
Stages	I–IV	Multiple subscores	I–III	I–IV
Ossicular assessment	Preoperative	Preoperative	Postoperative	Preoperative
Difficult access sites	-	S1, S2	Included	-
Ventilation/pneumatization	-	-	Included (E)	-
Inter-rater reliability	High	Moderate-high	Moderate-high	Not formally assessed
Recidivism prediction	Limited	Superior for recurrence	Limited	Moderate
Hearing prediction	Moderate	Ossicular subscore useful	O and L subscores useful	Limited
Target population	Acquired (adult)	Acquired (adult)	Acquired (adult)	Congenital (children)

## Imaging modalities for the evaluation of bone erosion in MEC

Current clinical practice relies on a combined evaluation using preoperative imaging and intraoperative direct observation. The assessment of bone erosion in MEC remains critically dependent on cross-sectional imaging, with high-resolution computed tomography (HRCT), magnetic resonance imaging (MRI), and diffusion-weighted imaging (DWI) serving as the primary modalities. Each technique offers distinct advantages but also carries inherent limitations that must be weighed in clinical decision-making.

HRCT remains the cornerstone of preoperative evaluation due to its superior spatial resolution and ability to delineate ossicular chain integrity, bony labyrinthine erosion, and extension into critical anatomical regions. Its wide availability and cost-effectiveness support its role as a first-line imaging tool in the preoperative setting [44–46]. A study evaluating CT-operative correlation found the strongest agreement for malleus erosion, followed by lateral semicircular canal and tegmen tympani involvement with weaker correlation for facial nerve dehiscence [47]. However, its limited soft-tissue contrast hinders the differentiation of cholesteatoma from adjacent tissues, posing a risk of missing early or residual disease. MRI offers superior soft-tissue contrast for delineating cholesteatoma and assessing intracranial complications, but its high cost and limited accessibility restrict its use. DWI has emerged as a valuable adjunct for distinguishing cholesteatoma from non-cholesteatomatous inflammatory tissue, offering high specificity in detecting residual or recurrent disease [48, 49]. However, although DWI is a non-invasive imaging modality, its limited sensitivity for lesions smaller than approximately 2–3 mm and inability to directly depict the extent of bony erosion limit its role as a standalone imaging modality. Another study has indicated that fusing Periodically Rotated Overlapping Parallel Lines with Enhanced Reconstruction (PROPELLER) DW-MRI with corresponding temporal bone CT images could improve the diagnostic and localization accuracy of cholesteatoma [50]. Collectively, no single technique comprehensively captures the full spectrum of cholesteatoma-induced bone destruction. This underscores the need for an integrated imaging strategy and highlights the ongoing demand for novel biomarkers or imaging paradigms capable of reflecting the underlying biological activity of the disease.

## Future directions

Although notable progress has been made in comprehending the biological mechanisms and clinical manifestations of MEC, substantial gaps still exist in precisely evaluating disease severity and forecasting clinical outcomes. Presently, owing to the fact that the grading of bone erosion severity is still predominantly in the exploratory stage, studies assessing bone erosion MEC display substantial heterogeneity. While some investigations prioritize ossicular chain integrity as the primary metric, others emphasize the extent of erosion and associated complication risk. This inconsistency hampers meaningful cross-study comparisons of clinical outcomes, including treatment efficacy and recurrence rates.

Future research should prioritize the development of integrated severity assessment frameworks that combine anatomical extent, bone erosion patterns, auditory function, and complication risk into unified prognostic models. Concurrently, quantitative imaging methodologies founded on HRCT and DWI, in conjunction with the radiomics techniques, have the potential to facilitate the objective characterization of bone destruction and enhance preoperative risk stratification. Another promising direction involves investigating quantitative biomarkers, such as Ki-67, MMPs, and RANKL, and their association with disease progression. In addition, the integration of artificial intelligence-assisted image analysis could further enhance diagnostic accuracy and support automated evaluation of erosion severity. Large-scale, multicenter studies employing standardized staging criteria and long-term follow-up are warranted to develop robust prognostic models for cholesteatoma recurrence. Established risk factors include patient age, disease extent (e.g., mastoid and ossicular chain erosion, lateral semicircular canal and facial nerve canal involvement), difficult-access sites (S1: supratubal recess; S2: sinus tympani), and surgical variables such as canal wall-up procedure and completeness of resection. Integrating these factors into multivariable prediction models will enhance risk stratification and inform individualized surgical planning.

The convergence of molecular biomarkers, AI-assisted imaging, and multivariable models promises to shift cholesteatoma assessment from descriptive staging toward biology-driven, individualized evaluation, ultimately enabling personalized surgical planning and improved outcomes.
